# Immersive Virtual Reality to Improve Outcomes in Veterans With Stroke: Protocol for a Single-Arm Pilot Study

**DOI:** 10.2196/26133

**Published:** 2021-05-10

**Authors:** Johanna E Tran, Christopher A Fowler, Jemy Delikat, Howard Kaplan, Marie M Merzier, Michelle R Schlesinger, Stefan Litzenberger, Jacob M Marszalek, Steven Scott, Sandra L Winkler

**Affiliations:** 1 Physical Medicine and Rehabilitation Service James A Haley Veterans' Hospital Tampa, FL United States; 2 Department of Internal Medicine Morsani College of Medicine University of South Florida Tampa, FL United States; 3 Research and Development Service James A Haley Veterans' Hospital Tampa, FL United States; 4 Department of Psychiatry and Behavioral Neurosciences Morsani College of Medicine University of South Florida Tampa, FL United States; 5 Advanced Visualization Center Information Technology and Research Computing University of South Florida Tampa, FL United States; 6 Department of Physical Medicine and Rehabilitation Morsani College of Medicine University of South Florida Tampa, FL United States; 7 Department of Psychology University of Missouri-Kansas City Kansas City, MO United States; 8 Polytrauma Rehabilitation Center James A Haley Veterans' Hospital Tampa, FL United States; 9 Department of Neurology Morsani College of Medicine University of South Florida Tampa, FL United States; 10 Department of Occupational Therapy Nova Southeastern University Fort Lauderdale, FL United States

**Keywords:** stroke, immersive virtual reality, feasibility, veterans affairs, veterans, pilot, recovery, upper extremity

## Abstract

**Background:**

Over the last decade, virtual reality (VR) has emerged as a cutting-edge technology in stroke rehabilitation. VR is defined as a type of computer-user interface that implements real-time simulation of an activity or environment allowing user interaction via multiple sensory modalities. In a stroke population, VR interventions have been shown to enhance motor, cognitive, and psychological recovery when utilized as a rehabilitation adjunct. VR has also demonstrated noninferiority to usual care therapies for stroke rehabilitation.

**Objective:**

The proposed pilot study aims to (1) determine the feasibility and tolerability of using a therapeutic VR platform in an inpatient comprehensive stroke rehabilitation program and (2) estimate the initial clinical efficacy (effect size) associated with the VR platform using apps for pain distraction and upper extremity exercise for poststroke neurologic recovery.

**Methods:**

This study will be conducted in the Comprehensive Integrated Inpatient Rehabilitation Program at the James A Haley Veterans’ Hospital. Qualitative interviews will be conducted with 10 clinical staff members to assess the feasibility of the VR platform from the clinician perspective. A prospective within-subject pretest-posttest pilot design will be used to examine the tolerability of the VR platform and the clinical outcomes (ie, upper extremity neurologic recovery, hand dexterity, pain severity) in 10 veteran inpatients. A VR platform consisting of commercially available pain distraction and upper extremity apps will be available at the participants’ bedside for daily use during their inpatient stay (approximately 4-6 weeks). Clinician interviews will be analyzed using qualitative descriptive analysis. Cohen *d* effect sizes with corresponding 95% CIs will be calculated for upper extremity neurologic recovery, hand dexterity, and pain. The proportion of participants who achieve minimal clinically important difference after using the VR platform will be calculated for each clinical outcome.

**Results:**

This study was selected for funding in August 2020. Institutional review board approval was received in October 2020. The project start date was December 2020. The United States Department has issued a moratorium on in-person research activities secondary to COVID-19. Data collection will commence once this moratorium is lifted.

**Conclusions:**

Our next step is to conduct a large multi-site clinical trial that will incorporate the lessons learned from this pilot feasibility study to test the efficacy of a VR intervention in inpatient rehabilitation and transition to home environments. When VR is used in patients’ rooms, it serves to provide additional therapy and may reduce clinician burden. VR also presents an opportunity similar to home-based practice exercises. VR can be implemented in both clinical settings and people’s own homes, where engagement in ongoing self-management approaches is often most challenging. This unique experience offers the potential for seamless transition from inpatient rehabilitation to the home.

**International Registered Report Identifier (IRRID):**

PRR1-10.2196/26133

## Introduction

### Background

Over the last decade, virtual reality (VR) has emerged as a cutting-edge technology in stroke rehabilitation. VR is defined as a type of user-computer interface that implements real-time simulation of an activity or environment, allowing user interaction via multiple sensory modalities [1]. VR interventions can be characterized as immersive or nonimmersive. Immersion refers to the sensation of being inside a particular environment or world, for example, a 3D world [2]. Nonimmersive VR typically uses commercial video game systems developed by the entertainment industry for home use, although some researchers have developed rehabilitation-specific nonimmersive VR apps [3-5]. Nonimmersive VR uses 2D interfaces such as Nintendo Wii, Microsoft Xbox, and Sony PlayStation [6-8]. Immersive VR uses a 3D virtual environment with the intention of making the user feel a part of, inside, or immersed in the environment to the extent that they become unaware of their physical surroundings [2]. Immersive VR experiences typically involve the use of a head-mounted display (HMD), which creates a 3D image in all fields of view. We will use the most current VR technology, which at this time is a wireless immersive HMD app with hand controllers, the Oculus Quest 2.

### Upper Limb VR Research

Upper limb deficits occur in up to 85% of stroke survivors and they significantly affect performance of activities of daily living [9]. The literature on the use of VR in stroke rehabilitation is fairly extensive, but is characterized by small, lesser quality studies with widely varying definitions of what constitutes a VR intervention. The stroke VR literature base has been criticized for lack of a control group, making it difficult to discern if positive effects were the result of the VR intervention itself or simply the result of extra therapy time, for example, when VR is used as an adjunct [10]. Studies on the use of VR for poststroke upper limb dysfunction have shown mixed results [2-8,11-15]. A Cochrane review published in 2017 [16] concluded that the overall effects of VR on upper extremity function were not significantly different when compared with those of conventional therapy (including both specialized VR systems designed for rehabilitation or commercial gaming consoles). However, when VR was utilized as an adjunct to standard care compared with no additional intervention (increased overall therapy time), the VR group experienced statistically significant benefits in upper limb function (standardized mean difference 0.49, 95% CI 0.21-0.77). The overall quality of the trials included for upper limb function outcomes is low. The Cochrane review also found a small, yet statistically significant effect of VR on activities of daily living (standardized mean difference 0.25, 95% CI 0.06-0.43). Because of the heterogeneity in the outcomes used in the studies investigating the effect of VR on upper limb function after a stroke, 2 systematic reviews and meta-analyses [1,17] grouped outcomes by the International Classification of Function domains. For studies that used a virtual world environment approach to VR, medium effect sizes were found: body structure/function effect size of 0.43 [1] to 0.54 [17], activity effect size of 0.54 [1] to 0.62 [17], and participation effect size of 0.38 [17] to 0.56 [1]. Gains after the intervention were preserved at follow-up [17]. A limitation of both systematic reviews and meta-analyses was the variability in how VR was delivered in terms of intensity and duration [1,17] and lack of clarity regarding control group therapy.

Three recent randomized controlled trials (RCTs) [10,18,19] of nonimmersive VR interventions (using 2D interfaces) that included control groups dose matched for therapy time found mixed results. A single-center study [19] that compared 10 sessions of a self-administered upper extremity rehabilitation program, including 4 game apps on a smartphone and tablet with control therapy of 1 hour of conventional occupational therapy per day found a significant difference on the Fugl-Meyer Assessment of Motor Recovery after Stroke-Upper Extremity (FMA-UE) at 1-month follow-up in favor of the intervention group. In contrast, neither the efficacy and safety of nonimmersive VR exercising in stroke (EVREST) rehabilitation trial [10] that compared 10 sessions of commercial gaming with control recreational activities or the VR training for upper extremity in subacute stroke multi-center trial [18] that compared 16 sessions of VR designed for rehabilitation with conventional therapy found significant differences. The authors of the EVREST study did, however, speculate that utilizing an immersive VR system might have led to significant results. As VR becomes more immersive, more interactive, and less expensive, and because of its flexibility, studies of the use of VR in the inpatient environment [20] suggest that VR is efficacious, easy to use, safe, and contributes to high patient satisfaction.

### VR and Pain

A recent multi-site study (N=546) found a 30% prevalence of pain across the acute, subacute, and chronic poststroke stages [21]. Cognitive factors (eg, attention) are important to pain perceptions, even when people are not engaged in specific tasks [22]. Theory suggests that VR directly or indirectly affects cognitive and attentional processes to attenuate pain. VR can be a distraction mechanism that consumes cognitive and attentional resources to limit pain-processing capabilities [23]. A randomized crossover study found a 56% reduction in time thinking about pain when using VR versus self-selected distraction (eg, meditation, smartphone; *P*<.001) [24]. VR may also create neurobiological interactions in the brain by regulating sensory stimulation to produce an analgesic effect [25]. Sense of immersion and presence are important to distraction and analgesia because distraction therapy is the most commonly used intervention in VR pain research [26]. A rapid evidence assessment of VR (20 studies, N=337) found strong evidence for short-term reduction in pain intensity and moderate evidence for pain analgesia [27]. A meta-analysis (14 studies, N=581) estimated a large, standardized effect (0.90, 95% CI 0.72-1.08) for VR pain distraction studies using between-group and mixed-model designs [28]. Thus, integration of VR during rehabilitation may have promising implications for poststroke pain.

### Neuroplasticity

Decades of animal research and recent research in human subjects provide compelling evidence that the adult brain affected by stroke can reorganize itself in response to experience and training, with sufficient repetition playing a critical role [29-31]. In patients with subacute stroke, gains in the upper limb and hand dexterity (strength, range of motion, speed of movement) require more intensive repetitive task practice than gains in lower limb and mobility [31-33]. In addition, task motivation is essential for learning [29,30,34-36]. Immersive VR exposure is hypothesized to deliver the crucial impetus to drive lasting neural changes by providing a motivating environment for poststroke patients to retrain movement, range of motion, movement speed, fractionation (use of individual fingers), and force production [37]. In the proposed study, immersive VR will be utilized as adjunct therapy, allowing patients to increase their therapy dose and thereby engage in the repetition essential for motor learning.

### Immersive VR

Nonimmersive VR environments are projected on 2D screens (eg, laptop). Nonimmersive VR can facilitate stroke symptom improvement [10,18,19], but it is lower on the immersion spectrum and less efficacious than immersive 3D VR [26,38]. Immersion and presence are theoretical mechanisms of change, which may facilitate greater learning within virtual environments [23,39]. Immersive VR interventions may be cost-effective and less resource-intensive than many traditional interventions with comparable efficacy [40].

### Preliminary Studies

In a pilot study [41,42] in the James A Haley Veterans’ Hospital (JAHVH) inpatient Chronic Pain Rehabilitation Program, our team found evidence for the feasibility of immersive VR within the chronic pain population as well as a decrease in fear of movement, pain interference with mobility, pain intensity, and pain catastrophizing. Veteran attendance (91%) and completion of attended 20-minute VR sessions was high (97%). Veterans typically rated 20-minute VR sessions as too short. According to the Department of Veterans Affairs (VA) Informatics and Computing Infrastructure (VINCI), in fiscal year 2018, there were more than 10,000 unique veteran inpatient admissions for stroke. The proposed study is an innovative treatment paradigm utilizing sophisticated immersive VR technology available at the bedside to increase therapy dosage. This cutting-edge technology has the potential to not only drive neurologic recovery by augmenting the brain’s own intrinsic repair capacity in response to a stroke insult (neuroplasticity) but also improve veterans’ quality of life by diminishing pain and enhancing self-efficacy. Immersive VR could ultimately become a new standard of care in acute inpatient rehabilitation, allowing unlimited rehabilitation experiences for patients with stroke. In addition, there is strong potential for seamless transition to home, as immersive VR technology rapidly becomes more sophisticated and less costly. Finally, the proposed research supports modernization of the veterans’ health administration by incorporating technology-assisted rehabilitation, addresses the VA Rehabilitation Research and Development (RR&D) goal of maximizing functional recovery, and focuses on VA Office of Research and Development priorities, including access to care, mental health, health care value, and pain.

### Aims and Research Questions

The proposed feasibility pilot project will address the RR&D goal of maximizing functional recovery by pilot testing an immersive VR intervention designed to increase exercise dosage for the upper limb and decrease pain for inpatient veterans after stroke without increasing therapist time [43]. The VR intervention will use an HMD, more commonly known as goggles, to which selected apps can be uploaded. Apps and goggles are commercially available and have been selected based on the following criteria: (1) address the treatment goals of overall upper extremity neurologic recovery, hand dexterity, and pain reduction, (2) utilized while patient lying in bed, (3) provide no stimulation to move legs or reach outside of bed area, (4) simple to use (require no technological expertise), (5) involve graded head, neck, upper extremity movement, and distraction to reduce pain, and (6) cognitive burden ranges from minimal to moderate. The VR intervention will be administered at bedside for two 30-minute therapy sessions per day for 4 weeks. The primary objective of this study is to determine the effectiveness of using VR as an adjunct to usual care therapy to enhance upper extremity neurologic recovery and hand dexterity and to decrease pain. Findings from this study will inform a larger multi-site RCT.

Our proposal is innovative in 4 distinct ways. First, we will use immersive 3D rather than the more typically used 2D VR. Immersion and the resulting “presence” within the virtual environment are thought to be the principal mechanisms of positive change [23,32]. Second, we will assess pain reduction after stroke by using VR apps, which is not well represented in the literature. Third, we are using VR as an adjunct therapy—adding additional therapy time with less burden on clinicians than is required in traditional therapy. Finally, VR when used in patients’ rooms presents an opportunity similar to home-based practice exercises. Our targeted enrollment is 10 clinical staff (research question [RQ] 1.1) and 10 inpatient veterans being treated for stroke (aim 2).

Specific aim 1: Determine the feasibility and tolerability of using a therapeutic VR platform in an inpatient comprehensive stroke rehabilitation program.

*RQ 1.1:* What is the feasibility of using the VR platform from the clinician perspective?

*RQ 1.2:* What is the tolerability for poststroke inpatients using the VR platform?

Specific aim 2: Estimate the initial clinical efficacy or effect size associated with the VR platform using apps for distraction and upper extremity exercise for veterans after the stroke.

*RQ 2.1:* What are the estimated effect sizes and degree of precision for the outcomes of upper extremity neurologic recovery, hand dexterity, and pain?

*RQ 2.2:* How clinically responsive are dexterity and upper extremity neurologic recovery (primary) outcomes to early stroke rehabilitation using a therapeutic VR platform?

## Methods

### Design

Our methodological framework is based on the work by the Virtual Reality Clinical Outcomes Research Experts committee [38]. We will use their VR2 clinical study design: conducting early prospective testing with a focus on feasibility and tolerability (aim 1) and initial efficacy (aim 2). Per Virtual Reality Clinical Outcomes Research Experts guidelines, we will use a single group so that we may optimize recruitment to represent the breadth and depth of our target patients.

### Population

There are 2 populations for the proposed project. The first population consists of veterans (n=10) who have been diagnosed with an acute ischemic or hemorrhagic stroke and are admitted to JAHVH inpatient rehabilitation after a stroke. Inclusion criteria are as follows: (1) age 18-80 years and (2) stroke diagnosis verified by brain imaging. Exclusion criteria are as follows: (1) unable to follow instructions or participate in immersive VR therapy due to significant cognitive impairment and (2) history of seizures. The second population consists of occupational therapists and rehabilitation nurses (clinician champions) working in the Comprehensive Interdisciplinary Inpatient Rehabilitation Program, who will provide data on the feasibility of using VR in an inpatient environment (RQ 1.1).

### Recruitment

All patients admitted to the Comprehensive Interdisciplinary Inpatient Rehabilitation Program at JAHVH (a designated Primary Stroke Center) with a diagnosis of acute ischemic or hemorrhagic stroke will be considered for inclusion in the study. A minimum of 5 beds will be designated for study participants. The Comprehensive Interdisciplinary Inpatient Rehabilitation Program admits 3.5 stroke patients per month, that is, 42 per year. We feel this is a sufficient subject pool from which to enroll the target sample size of 10 patients (16% of the patients admitted over the 18-month enrollment period). We have found that the technology is motivating for patients, which will help retention [42].

### Procedure

#### VR Intervention

**Figure 1:**
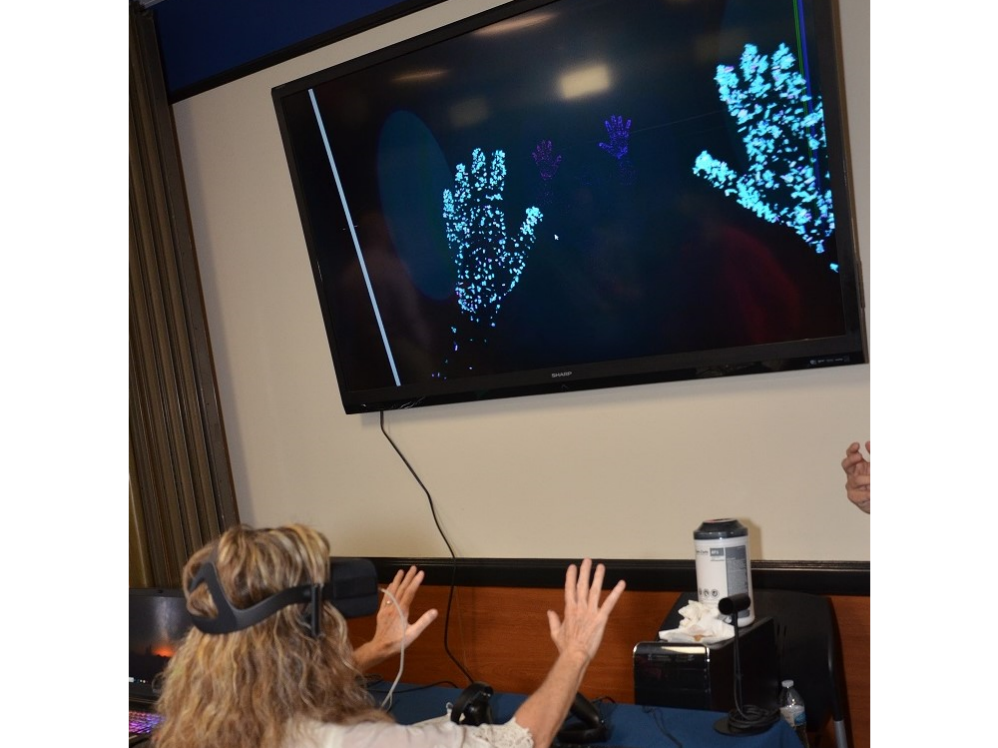
Virtual reality intervention.

**Figure 2:**
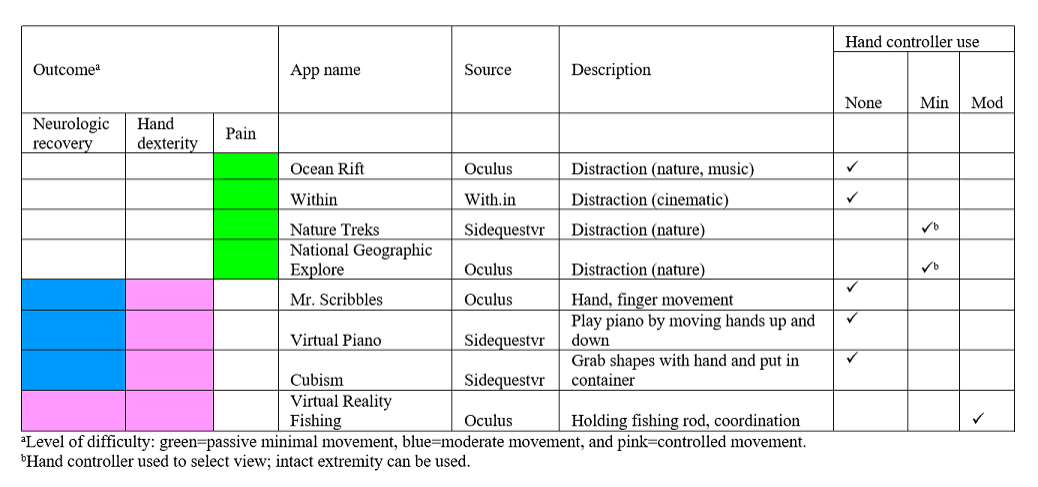
Apps in the virtual reality toolkit for Oculus Quest.

The VR intervention uses off-the-shelf technology: Oculus Quest HMD and commercially available apps specifically developed or adapted for Oculus Quest (Figure 1). App selection for individual patients will be guided by the motor difficulty of the apps (Figure 2). For example, patients will begin with the green-coded apps, which is the easiest activity level in the toolkit. These apps primarily address pain via distraction with minimal head and neck movement, but no hand movement, required. As tolerated, patients will advance to more difficult apps that require hand and finger movement, with high-level apps requiring controlled movement. Apps are commercially available and have been selected based on the following criteria: (1) address the treatment goals of overall upper extremity neurologic recovery, hand dexterity, and pain reduction; (2) can be utilized while patient is lying on bed; (3) provide no stimulation to move legs or reach outside of bed area; (4) are simple to use (require no technological expertise); (5) involve graded head, neck, upper extremity movement and distraction to reduce pain; and (6) cognitive burden ranges from minimal to moderate. Because hand-tracking app technology is developing/improving at a rapid pace, upon notice of funding, it is likely that we will need to update the VR toolkit (Figure 2).

Prior to beginning the intervention, clinician champions (occupational therapists and nurses) and the project manager will be instructed with regard to the use of the HMD and VR apps by our technologist. Staff will have the opportunity to practice with the HMD and apps for 2 weeks prior to using the apps with patients. Following institutional review board approval and funding on site, potential subjects will be identified by the admitting physician, principal investigator, or the project manager in the absence of the principal investigator. The project manager will use a Health Insurance Portability and Accountability Act waiver to check the inclusion and exclusion criteria.

#### Week 1: Baseline and Preintervention Data Collection

Once patients are enrolled, the project manager will collect the baseline data and administer the preintervention outcome measures (Table 1). Further, the principal investigator/Dr Tran, occupational therapists, project manager, and the technologists will select apps from the VR toolkit (Figure 2) that best address the individual patient’s treatment goals based on his/her current functional level.

**Table 1:** Outcome variables and covariates.

Variable		Definition
**Outcomes^a^**		
	Hand dexterity (primary measure)	Action Research Arm Test (MCID^b^: chronic=5.7, acute=12)
	Neurologic recovery (primary measure)	Fugl-Meyer Assessment of Motor Recovery after Stroke-Upper Extremity (MCID 4-7)
	Pain (secondary measure)	Pain Outcomes Questionnaire-Veterans Affairs, initial, item 12 and discharge, item 2 (pain numeric rating scale) (effect size 0.85, medium effect, standard error of measurement 0.79)
**Demographic and clinical^c^**		
	Age	Age on date of baseline data collection
	Sex	Male/female
	Race/ethnicity	Caucasian, African American, Hispanic, other
	Time since index stroke	In days: index event – baseline data collection
	Type of stroke	Ischemic=0, hemorrhagic=1

^a^Source of information from clinical assessment and self-report.

^b^MCID: minimal clinically important difference.

^c^Source of information from computerized patient record system.

#### Weeks 2-4: VR Intervention

Patients will be instructed in the use of the HMD with VR apps by a project occupational therapist. It is anticipated that subjects may need 1-3 sessions of instruction. VR dosage will be 2 half-hour sessions per therapy day, facilitated by an occupational therapist and clinician champions, overseen by the principal investigator. The timing of VR sessions will vary based on the patient’s therapy schedule. During the VR session, the patient will be reclining or seated in bed with both bed rails raised. The clinician champions will bring the VR HMD to the bedside and assist the patient with donning the device. Once the patient is comfortable using the HMD with VR apps, the clinician champion will begin each session by setting the patient up and making sure that they are successfully engaging with the app. The clinician champion will return 30 minutes later to remove the VR HMD from the room. This process will be repeated a second time each therapy day. Patients can initiate the use of a more challenging app (blue category) that gradually includes hand/arm movement. Some patients may progress to the pink category in which hand/arm coordination is required.

##### Week 4: Postintervention Data Collection

The average length of stay in the acute inpatient rehabilitation unit at JAHVH is 4-6 weeks. Accordingly, postintervention data will be collected at week 5 or at the end of week 4 if the veteran is being discharged. RQ 1.2 tolerability data will be collected throughout the subjects’ participation in the study.

#### End of Data Collection

Once all veterans have completed the study, RQ 1.1 feasibility data will be collected from clinician champions.

### Outcomes

#### Aim 1

Feasibility is the degree to which the VR treatment can be successfully integrated within the flow of usual care [13]. Feasibility will be measured with a 6-item survey based on the Consolidated Implementation Framework [44] that will be administered to 10 clinical staff using Research Electronic Data Capture (REDCap). Tolerability refers to the prevalence of patient-reported physical (eg, vertigo, nausea, cybersickness) and emotional (eg, fear, anxiety) adverse effects of the VR treatment, along with any discomfort or inconvenience related to the VR equipment (eg, ill-fitting headset, facial discomfort, inability to explore the 3D environment fully due to limited mobility) [32,38]. Tolerability data (complaints and adverse events frequencies) will be extracted from detailed meeting minutes where such events are reported and discussed.

#### Aim 2

##### Primary Measures

Hand dexterity will be measured using the Action Research Arm Test [45,46]. The 19-item Action Research Arm Test is a validated assessment of upper extremity limitations across 4 activity subdomains as rated by a clinician: grasp, grip, gross movement, and pinch [45]. Items are summed for each subscale with higher scores indicating more normal levels of functioning. The minimal clinically important difference (MCID) for chronic pain is a 5.7-point reduction from baseline [45,46]. Neurologic recovery will be measured using the FMA-UE [47]. The FMA-UE is a clinician-administered assessment of impairment in upper extremity motor functioning across multiple domains, including upper extremity, wrist, hand, and coordination/speed. Items are summed for each subscale with higher scores indicating greater improvement in functioning. The MCID for the FMA-UE subscales is 4.25-7.25–point reduction from the baseline [47].

##### Secondary Measures

The Intake and Discharge Questionnaires from the Pain Outcomes Questionnaire-VA (POQ-VA) will be utilized to assess pain-related treatment outcomes [48]. Specifically, we will use a pain numeric rating (intake item 12, discharge item 2) scale of 0 (no pain at all) to 10 (worst possible pain). Identical pain numeric rating scales are well-validated in the literature, but we were unable to identify the MCID for pain within a poststroke population.

### Analyses

A data set was used during the first month of the study by using Microsoft Excel software as Excel is easily imported into the statistical analysis system for analysis. We have chosen to use Excel on our local research server rather than VINCI because this is a prospective cohort of new admissions and a relatively small sample. Data will be collected and entered into the database by the project manager. Data entry will be verified by the principal investigator. Data will be stored on the secure JAHVH Research Service R-drive. With the proposed pilot study design, the overall analytic goals are to (1) determine the feasibility and tolerability of using a therapeutic VR platform in an inpatient comprehensive stroke rehabilitation program and to (2) estimate, with reasonable precision, the effect sizes of upper extremity neurologic recovery, hand dexterity, and pain reduction outcomes.

#### Aim 1

Qualitative descriptive analyses [49] will be used to address RQ 1.1 (feasibility) and RQ 1.2 (tolerability). For RQ 1.1, responses will be downloaded from REDCap. The 6 survey items address 3 feasibility constructs: adaptability, patient need, and staff comments. Responses for each construct will be pasted into an excel spreadsheet—one tab for each construct. Responses will then be grouped by similar content. Results will be reported as themes and subthemes. Similarly, for RQ 1.2, patient concerns, complaints, and adverse events associated with use of the VR platform will be abstracted from the research team meeting notes and will be tabulated. Responses will then be grouped by similar content. Results will be reported as themes and subthemes. Note that all adverse events will be immediately reported per VA and institutional review board policy. The analyses described here are for dissemination purposes.

#### Aim 2

For RQ 2.1, the primary outcomes will consist of preintervention to postintervention changes on 2 physical measures of stroke recovery: the Action Research Arm Test [45] and the FMA-UE [47]. Both of these measures are scored on a continuous scale, as is the outcome of pain, as listed in Table 1. Therefore, the initial step will be to examine the distributions of each outcome measure, including the distribution in the change of scores from preintervention to postintervention. To estimate effect sizes over 4 weeks with the use of the VR platform, standardized effect sizes and 95% CIs will be calculated using the within-group pretest/posttest design described by Morris and DeShon [50] and Kadel and Kip [51]. Considering that this is a pilot study design, which can have a potential type I error due to multiple outcomes evaluated, the confidence intervals for the 2 coequal primary outcomes will be evaluated with a type I error rate of 0.025 (ie, to determine if the confidence interval for the outcome difference scores includes the null effect size value of 0); secondary outcomes will be evaluated with a type I error rate of 0.01. The above confidence interval approach parallels the use of a paired two-sided *t* test to determine statistical significance.

For RQ 2.1, since the effect sizes to be calculated are standardized measures, corresponding results across these outcomes will be directly comparable. However, these metrics do not necessarily translate to meaningful clinical differences (improvements). Therefore, for those outcome measures with published metrics for MCID [52], results of the VR platform will be compared across outcomes. As listed in Table 1, the measures of dexterity and neurologic recovery have published references for MCID, whereas we are unaware of a published MCID for POQ-VA. Therefore, for POQ-VA, we will first determine the change (prescores versus postscores) in standard deviation units (from the baseline value) that denotes MCID for the measures of dexterity and neurologic recovery. We will then average these 2 calculations of standard deviation units to estimate the magnitude of change in prescores to postscores on the POQ-VA that may approximate MCID on this measure. Thus, in addition to the comparison of standardized effect sizes across the 3 outcomes measures, all 3 measures will be compared in terms of proportion of subjects who experience MCID.

## Results

**Figure 3:**
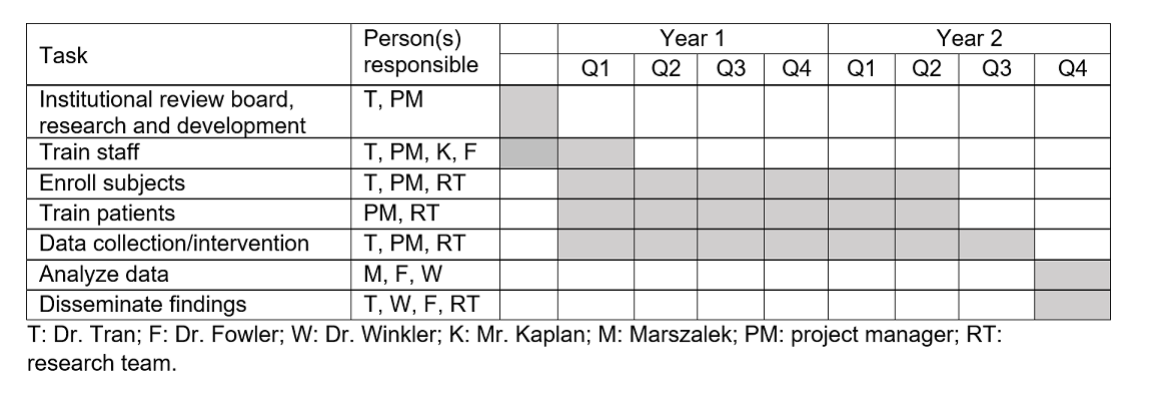
Study timeline.

This study was selected for funding by VA RR&D in August 2020. The approval for the study from the University of South Florida Institutional Review Board and the JAHVH R&D Committee (Protocol STUDY001075) was received in October 2020. The project start date was December 2020. All VR equipment for this study has been purchased and inventoried. Clinical staff are currently being trained to use the VR equipment in the clinic. The United States Veterans Health Administration has issued a moratorium on all in-person VA research activities secondary to COVID-19. Data collection will commence once this moratorium is lifted and will follow the projected study timeline presented in Figure 3.

## Discussion

### Overview of This Study

If the aims of this research are achieved, VR will be used in combination with established pain management strategies to improve neurologic recovery and hand dexterity and to decrease pain. The short-term goal of this project is to determine the feasibility of conducting an RCT to determine the effectiveness of using VR as an adjunct to usual care therapy to enhance upper extremity neurologic recovery and hand dexterity and to decrease pain. Our long-term goal is to provide veterans with an exercise and pain reduction modality that can serve as an adjunct to scheduled therapy and assist with the clinic to home transition. VR has the advantage of being easily implemented both within VA health care settings as well as veterans’ own residences, where engagement in ongoing self-management approaches is often most challenging [32,42].

### Potential Limitations and Strategies

As this pilot study will employ a within-subject design to evaluate the magnitude of stroke rehabilitation over 3 weeks with the use of VR technology, there will be no control condition to judge rehabilitation results to that which might be expected from time alone and natural history of stroke recovery. Therefore, as described for RQ 2.1, we will place a premium on evaluating rehabilitation results by using MCID, which is highly relevant to patients and generally would not be expected to be achieved simply from time alone (4 weeks).

### Dissemination

Dissemination will be led by the principal investigator. Channels for dissemination include (1) annual progress and final summary reports to VA RR&D service, (2) bulleted briefings to our Program Partner, (3) presenting findings at national and local research meetings/conferences and VA cyberseminars and Military Health System Speaker series, and (4) submitting manuscripts to relevant peer-reviewed journals.

### Conclusion

Examining the feasibility of this immersive VR intervention will be beneficial for veterans, clinicians, and policy makers. The health care market size of extended reality (ie, VR, augmented reality) technology utilization is projected to grow from nearly US $2.1 billion in 2019 to roughly US $8-11 billion by 2026-27 [53,54]. Despite this exceptional growth, published VR research to date often does not extend beyond pilot trials and case studies. Given the lack of large-scale RCTs examining the clinical effectiveness of immersive VR for poststroke rehabilitation, evidence from this pilot trial presents a key step to inform a larger multi-site trial.

## Abbreviations

EVREST: Efficacy and safety of nonimmersive Virtual Reality Exercising in Stroke
FMA-UE: Fugl-Meyer Assessment of Motor Recovery after Stroke-Upper Extremity
HMD: head-mounted display
JAHVH: James A Haley Veterans’ Hospital
MCID: minimal clinically important difference
POQ-VA: Pain Outcomes Questionnaire-Veterans Affairs
RCT: randomized controlled trial
REDCap: research electronic data capture
RQ: research question
RR&D: rehabilitation research and development
VA: Veterans Affairs
VINCI: Veterans Affairs Informatics and Computing Infrastructure
VR: virtual reality
